# Comparison of ASL and DSC perfusion methods in the evaluation of response to treatment in patients with a history of treatment for malignant brain tumor

**DOI:** 10.1186/s12880-024-01249-w

**Published:** 2024-03-22

**Authors:** Ezgi Suat Bayraktar, Gokhan Duygulu, Yusuf Kenan Çetinoğlu, Mustafa Fazıl Gelal, Melda Apaydın, Hülya Ellidokuz

**Affiliations:** 1 Department of Radiology, University of Izmir Katip Çelebi, Atatürk Training and Research Hospital, Izmir, 35360 Türkiye; 2 Department of Radiology, Manisa State Hospital, Manisa, 45130 Türkiye; 3 Department of Biostatistics and Medical Informatics, University of Dokuz Eylül, İzmir, 35340 Türkiye

**Keywords:** Perfusion MRI, Arterial spin labeling, Dynamic susceptibility contrast-enhanced, Glioblastoma, Metastasis

## Abstract

**Objective:**

Perfusion MRI is of great benefit in the post-treatment evaluation of brain tumors. Interestingly, dynamic susceptibility contrast-enhanced (DSC) perfusion has taken its place in routine examination for this purpose. The use of arterial spin labeling (ASL), a perfusion technique that does not require exogenous contrast material injection, has gained popularity in recent years. The aim of the study was to compare two different perfusion techniques, ASL and DSC, using qualitative and quantitative measurements and to investigate the diagnostic effectiveness of both. The fact that the number of patients is higher than in studies conducted with 3D pseudo-continious ASL (pCASL), the study group is heterogeneous as it consists of patients with both metastases and glial tumors, the use of 3D Turbo Gradient Spin Echo (TGSE), and the inclusion of visual (qualitative) assessment make our study unique.

**Methods:**

Ninety patients, who were treated for malignant brain tumor, were enrolled in the retrospective study. DSC Cerebral Blood Volume (CBV), Cerebral Blood Flow (CBF) and ASL CBF maps of each case were obtained. In qualitative analysis, the lesions of the cases were visually classified as treatment-related changes (TRC) and relapse/residual mass (RRT). In the quantitative analysis, three regions of interest (ROI) measurements were taken from each case. The average of these measurements was compared with the ROI taken from the contralateral white matter and normalized values (*n*) were obtained. These normalized values were compared across events.

**Results:**

Uncorrected DSC normalized CBV (nCBV), DSC normalized CBF (nCBF) and ASL nCBF values of RRT cases were higher than those of TRC cases (*p* < 0.001). DSC nCBV values were correlated with DSC nCBF (r: 0.94, *p* < 0.001) and correlated with ASL nCBF (r: 0.75, *p* < 0.001). Similarly, ASL nCBF was positively correlated with DSC nCBF (r: 0.79 *p* < 0.01). When the ROC curve parameters were evaluated, the cut-off values were determined as 1.211 for DSC nCBV (AUC: 0.95, 93% sensitivity, 82% specificity), 0.896 for DSC nCBF (AUC; 0.95, 93% sensitivity, 82% specificity), and 0.829 for ASL nCBF (AUC: 0.84, 78% sensitivity, 75% specificity). For qualitative evaluation (visual evaluation), inter-observer agreement was found to be good for ASL CBF (0.714), good for DSC CBF (0.790), and excellent for DSC CBV (0.822). Intra-observer agreement was also evaluated. For the first observer, good agreement was found in ASL CBF (0.626, 70% sensitive, 93% specific), in DSC CBF (0.713, 76% sensitive, 95% specific), and in DSC CBV (0.755, 87% sensitive - 88% specific). In the second observer, moderate agreement was found in ASL CBF (0.584, 61% sensitive, 97% specific) and DSC CBF (0.649, 65% sensitive, 100% specific), and excellent agreement in DSC CBV (0.800, 89% sensitive, 90% specific).

**Conclusion:**

It was observed that uncorrected DSC nCBV, DSC nCBF and ASL nCBF values were well correlated with each other. In qualitative evaluation, inter-observer and intra-observer agreement was higher in DSC CBV than DSC CBF and ASL CBF. In addition, DSC CBV is found more sensitive, ASL CBF and DSC CBF are found more specific for both observers. From a diagnostic perspective, all three parameters DSC CBV, DSC CBF and ASL CBF can be used, but it was observed that the highest rate belonged to DSC CBV.

## Introduction

Brain and other central nervous system (CNS) tumors are the most common cancer in children aged 0–14 years, the second most common cancer in adolescents aged 15–19 years, and the 8th most common cancer in adults over 40 years old [1]. In the United States, malignant brain and other CNS tumors are the 6th most common cause of cancer death in adults over 40 years of age [2]. Glioblastomas and brain metastases, which are the most common malignant tumors of the CNS, are associated with high recurrence and mortality rates, and their incidence has increased in recent years [3]. Surgery, radiotherapy (RT) and chemotherapy are used in combination in the standard treatment of malignant brain tumors. However, surgery generally cannot achieve total resection of the tumor. Therefore, high-grade glial tumors and metastatic tumors may recur after treatment [4]. For this reason, magnetic resonance imaging (MRI) is used as the standard imaging method in evaluating the response to treatment after surgery [5].

Conventional MRI shows anatomical structures and major changes in the structure of the tumor. However, in recent years, advanced MRI techniques have made it easier to distinguish physiological properties of tissues such as cellularity, metabolism and vascularity [6]. Perfusion MRI or perfusion-weighted imaging (PWI) techniques track the passage of specific particles through the microvascular bed, providing information on hemodynamic parameters such as CBF, CBV and mean transit time (MTT). Perfusion MRIs are advanced tools that have recently been used to differentiate both primary and metastatic brain tumors and include dynamic contrast-enhanced (DCE) MRI, DSC MRI, and ASL MRI [7, 8].

DSC perfusion is a perfusion method using exogenous contrast material (gadolinium [Gd] particles) and is frequently used in neuro-oncology, prognosis and evaluation of response to treatment [9]. The disadvantages of DSC perfusion are that it can be affected by susceptibility artifacts, its use is limited in children and people with renal failure due to the use of contrast material and the difficulty of exact quantification [9, 10]. ASL, a perfusion technique that does not require exogenous contrast material injection, uses endogenous intravascular tracer-hydrogen nuclei in the blood [11]. ASL, which has high sensitivity in evaluating microvascular proliferation, allows the measurement of CBF values, thus allowing exact quantification and healthy comparison [10, 12]. Disadvantages of ASL include low signal-to-noise ratio, sensitivity to motion and susceptibility artifacts, slower acquisition time, affected by hydrocephalus and sedation selection [13]. Previous studies have shown that there is a close linear correlation between ASL and DSC-MRI results [7, 14–16]. The aim of our study is to compare the effectiveness of ASL perfusion, which is a non-contrast option, with DSC perfusion in evaluating tumor follow-up cases, along with technological developments in recent years.

The fact that the number of patients is higher than in studies conducted with 3D pCASL, the study group is heterogeneous as it consists of patients with both metastases and glial tumors, the use of 3D TGSE, and the inclusion of visual (qualitative) assessment make our study unique.

## Materials and methods

### Patients

Patients who underwent ASL and DSC perfusion MRI between June 2022 and January 2023 were enrolled in this retrospective study. The study received ethics committee approval from Izmir Katip Çelebi University non-invasive clinical research committee (application number 2022-GOKAE-0712, dated 22/12/2022, Decision no: 0617). Criteria for inclusion in the study were determined as being diagnosed with a malignant brain tumor, having received surgery and/or radiotherapy treatment for the defined lesion, and having had DSC and ASL MRI scans. Exclusion criteria from the study were that the lesion was benign or had no mass, there was no history of surgery and/or RT, and MRI sections were not of technical quality that could be evaluated.

Of the 166 images obtained between June 2022 and January 2023, 20 were excluded from the study because they were repeats, 8 patients did not have a mass or were benign, ASL or DSC perfusion images were not of appropriate quality in 22 patients, 7 patients had not started treatment, and the lesions in 9 patients were too close to the vascular structures or too small to be measured in size. A total of 90 patients with malignant brain tumors (primary or metastatic) were included in the study. In cases with more than one lesion, the largest treated lesion was included in the analysis.

### Magnetic resonance imaging acquisition

All imaging was performed on a 3 T MRI unit (Siemens Healthlineers, Magnetom Lumina) with a 32-channel head coil while the patient was in the supine position. Evaluation and processing of images and creation of perfusion maps were carried out via the workstation (Syngo.via serial number: 221348, Siemens Healthlineers). The sequence parameters used were hardcoded and cannot be changed by users. These parameters in MRI acquisition are listed in Tables 1 and 2. Contrast injection was performed after non-contrast MRI images and 3D TGSE, pCASL images were obtained. Before pCASL examination, presaturation was provided to eliminate the signal of stationary tissues. Then, labeling was made with a series of 28 degree RF pulses and the labeled protons were waited to arrive in the imaging area after the delay period. Images were taken at 16 different post-labeling delays in 24 different slices. For quantification, general kinetic model proposed by Buxton et al. [17, 18] is used (Eq. 1). Blood T1 was 1,66 s and labeling efficiency was assumed to be 0.8 [19].

1 $${\Delta \textrm{M}}_{\textrm{tiss}}=\left\{\begin{array}{ll}0& \textrm{t}< \textrm{ATT}\\ {}2{\textrm{M}}_{0,\textrm{b}}{\upalpha \textrm{T}}_{1,\textrm{app}}{\textrm{fe}}^{\hbox{-} \frac{\mathrm{ATT}}{{\textrm{T}}_{1,\textrm{blood}}}}\left(1\hbox{-} {\textrm{e}}^{\hbox{-} \frac{\textrm{t-ATT}}{{\mathrm{T}}_{1,\mathrm{app}}}}\right)& \textrm{ATT}\le \textrm{t}\le \textrm{ATT}+\uptau \\ {}2{\textrm{M}}_{0,\textrm{b}}{\upalpha \textrm{T}}_{1,\textrm{app}}{\textrm{fe}}^{\hbox{-} \frac{\textrm{ATT}}{{\mathrm{T}}_{1,\mathrm{blood}}}}{\textrm{e}}^{\hbox{-} \frac{\textrm{t-} \uptau \textrm{-ATT}}{{\mathrm{T}}_{1,\mathrm{app}}}}\left(1{\textrm{- e}}^{\hbox{-} \frac{\uptau}{{\textrm{T}}_{1,\textrm{app}}}}\right)& \textrm{ATT}+\uptau < \textrm{t}\end{array}\right. ,$$

**Table 1:** Sequence parameters used in the study

Sequence	TR	TE	Matrix	Section thickness (mm)	Section range (mm)	FOV
T2	4290	97	912 × 896	3	3.9	234 × 230
FLAIR	9000	98	464 × 512	3	3.9	199 × 220
T1 3D	2200	3.4	256 × 256	0.9	–	230 × 230
Postcontrast T1- 3D	2200	3.4	256 × 256	0.9	–	230 × 230
T2* perfusion (DSC)	3040	30	128 × 128	3	3.9	199 × 220
ASL	4420	22.3	128 × 128	4	–	220 × 220

**Table 2:** Multiphase pCASL image parameters

Maximum Labeling Duration	1800 ms
Inversion Time	800, 900, 1000, 1100, 1200, 1400, 1600, 1800, 2000, 2300, 2600, 2900, 3200, 3500, 3800, 4000 ms
Inversion Array Size	16
Turbo Factor	15
Flip Angle	28 degrees
Time Of Review	4:59 minutes

In DSC perfusion, 0.1 mmol/kg Gd contrast material was used and approximately ½ of the total dose (15 cc) was given as a saturation dose. After the saturation dose, 5 minutes were waited and the remainder of the contrast was injected with the injection pump at a rate no slower than 5 ml/min. Afterwards, 20–25 ml physiological saline was injected. Contrast injection was made 20 seconds after the MRI images were started in cine mode, and images began to be taken with GRE EPI T2* sequences with a temporal resolution of 2 seconds.

In the post-processing phase, standard Singular Value Decomposition (sSVD) was used for quantificiation [20]. After detecting the appropriate lesion on the workstation, local AIF was determined and the intensity-perfusion slopes of the selected cerebral artery were obtained. Perfusion maps were created from these intensity-perfusion slopes, and in addition to the raw images, fusion images were obtained by combining them with the best sequence in which the lesion could be observed.

### Image analysis evaluation process

#### Quantitative assessment

**Fig. 1:**
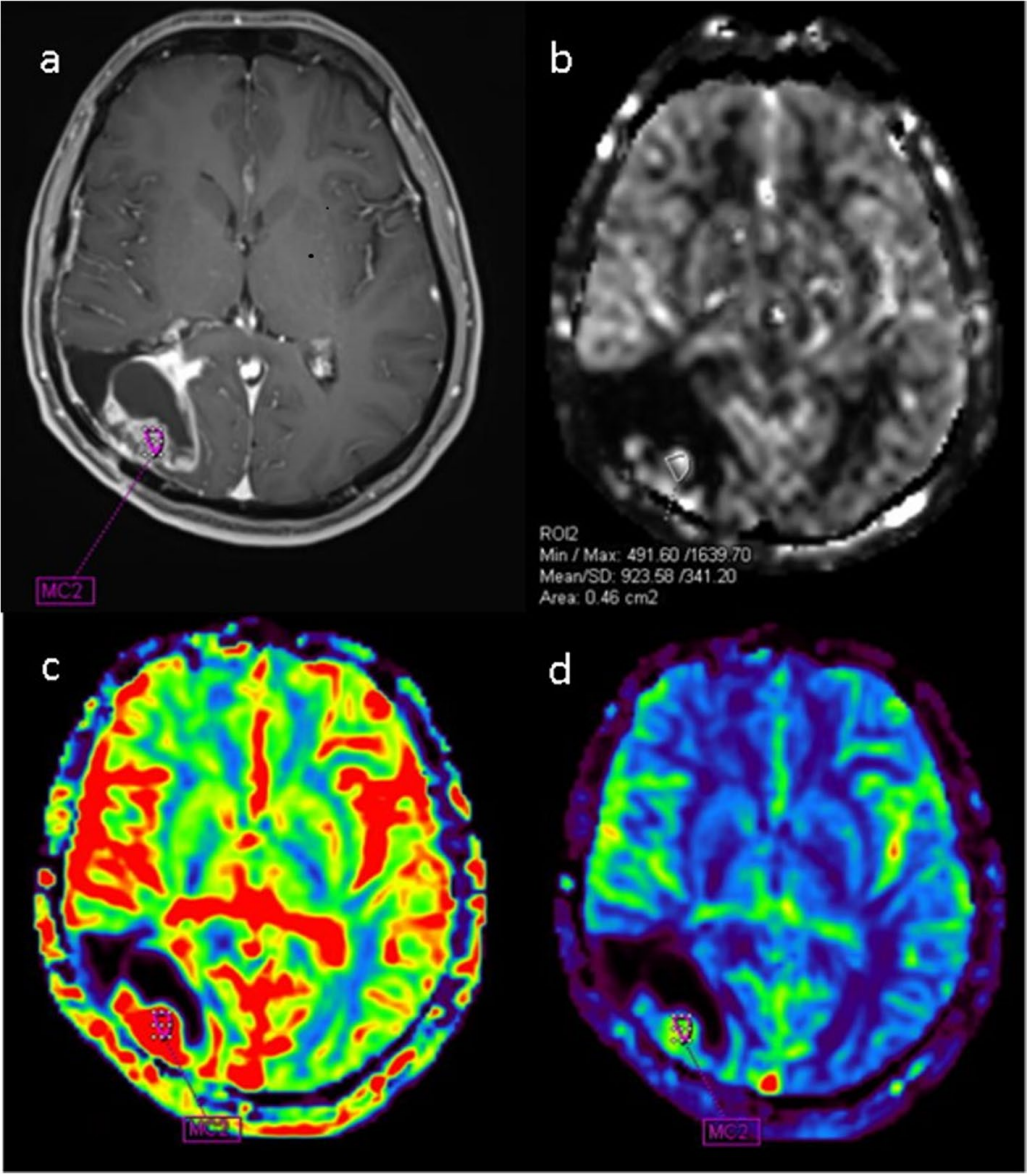
Case with a history of surgery and RT due to Grade 3 Astrocytoma; **a** Contrast-enhanced fat-suppressed T1: A solid-appearing area showing enhancement on the cavity wall after the operation. **b** ASL CBF: Hyperperfusion in the contrast-enhanced area. **c** DSC CBV: Marked hyperperfusion in the contrast-enhanced area. **d** Hyperperfusion is observed in the contrast-enhanced area. Categorized as non-residual tumor (NRT) based on follow-up imaging and clinical findings

The obtained images were processed and evaluated on the appropriate workstation (Syngo.via serial number: 221348, Siemens Healthlineers). CBF and CBV measurements on DSC perfusion maps were made from the most hyperperfused areas of the solid lesion, avoiding necrotic areas and vascular structures. If no perfused area was detected in the lesion, ROI measurements were made from the walls of the operation cavity or the solid part of the non-perfused lesion. The selected ROI sizes were chosen differently for each patient depending on the width of the recurrent tumor or the size of the size of the walls of operation cavity (Fig. 1). To avoid bias, 3 regions of interest (ROI) were placed and uncorrected CBF and CBV were measured with the free hand technique. Then, the average ROI value was obtained by taking the average of these 3 ROI values. Additionally, a reference ROI value was taken in the contralateral cerebral white matter for comparison. In cases where the contralateral white matter was not normal or the lesion was located in the posterior fossa, a reference ROI was taken from the contralateral brain parenchyma. The average ROI value was divided by the reference ROI value to create a standard normalized value (n) for the selected lesion for each patient.

Similarly, in ASL perfusion maps, CBF measurement was made from the hyperperfused sections if there was a lesion, from the solid section of the lesion if there was a lesion but hypoperfused, and from the walls of the operation cavity if the patient was operated on or there was no identifiable lesion, three ROI measurements were made using the free hand technique. These ROI measurements were averaged and the average ROI value was obtained.

**Fig. 2:**
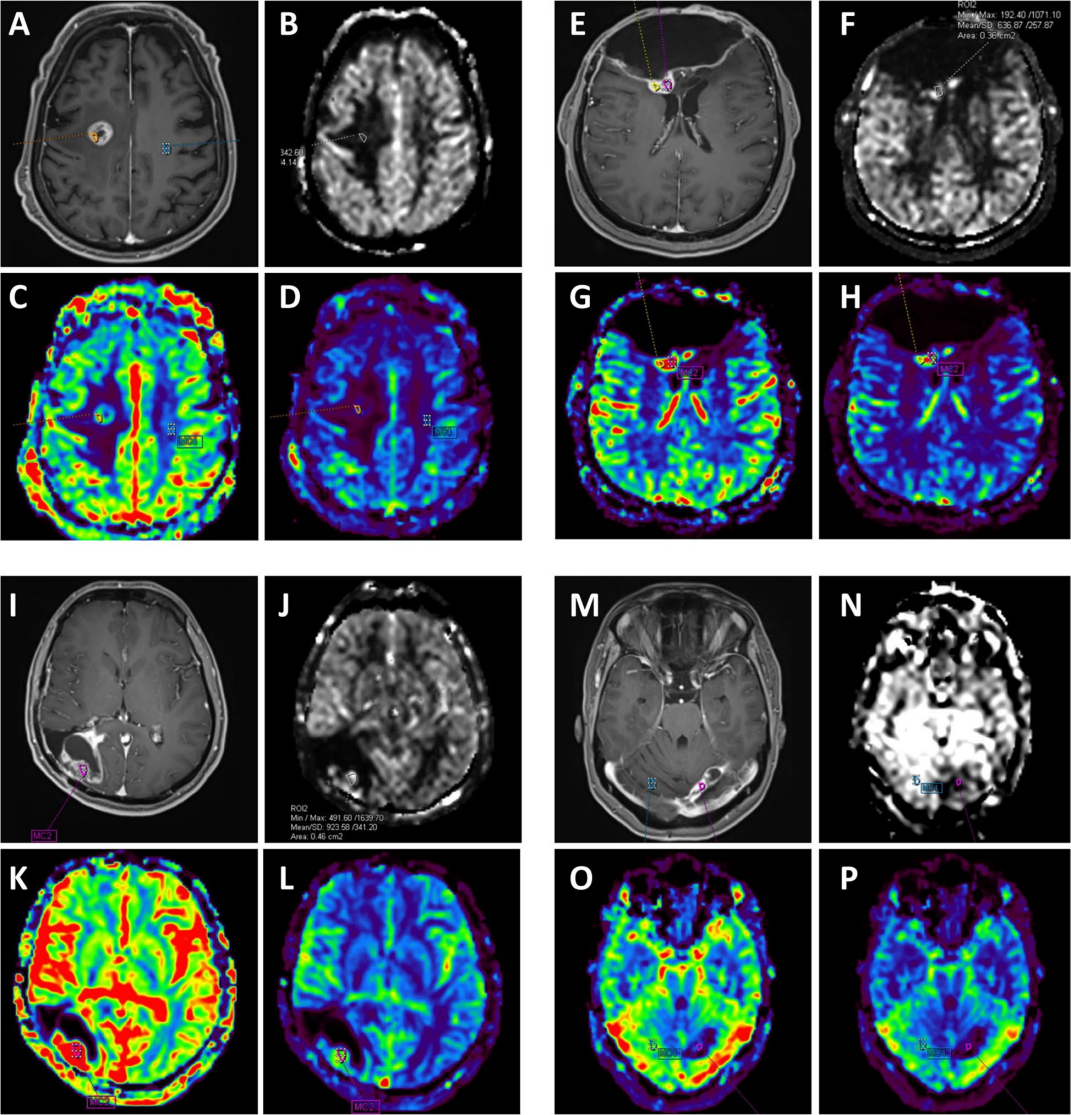
Post-RT evaluation in a patient with lung cancer and brain metastasis; **A** Contrast-enhanced fat-suppressed T1: metastatic lesion showing contrast enhancement, **B** ASL CBF: minimal signal change in the lesion site, **C** DSC CBV: Significant hyperperfusion in the lesion site, **D** DSC CBF: Mild moderate hyperperfusion is observed compared to CBV. It is categorized as RRT with follow-up imaging and clinic. Control image with recurrence of leiomyosarcoma metastasis and after surgery and RT history, **E** Contrast-enhanced fat-suppressed T1: Solid lesion with contrast enhancement in the posterior part of the operation cavity wall, **F** ASL CBF: Hyperperfusion in the lesion site, **G** DSC CBV: Marked hyperperfusion in the lesion site, **H** DSC CBF: Hyperperfusion in the lesion site. It is categorized as RRT with follow-up imaging and clinic. Case with a history of surgery and RT due to Grade 3 Astrocytoma, **I** Contrast-enhanced fat-suppressed T1: Solid-looking area showing contrast enhancement on the operation cavity wall, **J** ASL CBF: Hyperperfusion in the contrasted area, **K** DSC CBV: Significant hyperperfusion in the contrasted area, **L** Hyperperfusion is observed in the contrasted area. It is categorized as RRT with follow-up imaging and clinic. Control MRI in the case with a history of surgery and RT due to breast cancer brain metastasis, **M** Contrast-enhanced fat-suppressed T1: Moderate thickening of the wall medial to the operation cavity is observed in the left cerebellar hemisphere, **N** ASL CBF, **O** DSC CBV, **P** DSC CBF: The part medial to the cavity is hypoperfused compared to the opposite hemisphere. When evaluated with follow-up findings, it is in favor of TCR

The reference ROI value was taken from the opposite white matter. An attempt was made to create a normalized value by dividing the average ROI value by the opposite white matter ROI value (Fig. 2).

#### Qualitative assessment

Among conventional examinations, only contrast-enhanced T1 and FLAIR images were evaluated for each case. Colored CBF and CBV maps in DSC perfusion, and colorless and colored CBF maps in ASL perfusion were evaluated visually. Only the current images of the cases were evaluated without looking at their old images, and the scoring was carried out as follows:

- 0, Cases where no hyperperfused area is observed and only treatment-related changes (TRC) (such as radiation necrosis, operation cavity).
- 1, Monitoring of the hyperperfused area and/or residual or recurrent tumor (RRT) (recurrent tumor in the operating cavity, postoperative residual tumor, recurrent tumor).

In the first stage, the DSC CBV and CBF maps of the cases were visually evaluated. To avoid bias, readers evaluated ASL CBF values after 4 weeks. The evaluations were made separately by two radiologists with 18 years (G.D.) and 7 years (Y.K.C.) experience.

#### Analysis of data

In the current examination of cases, the gold standard technique for distinguishing between TRC and RRT is histopathological diagnosis with biopsy. However, biopsy is not a method that can be applied continuously in case follow-up. Therefore, for the outcome evaluation, the patient’s previous imaging, clinical findings and follow-up imaging were evaluated, and the character of the lesion in the current examination was determined accordingly.

### Statistical analysis

SPSS 27.0 program was used for statistical analysis. Three ROI measurements were made on the DSC CBV, CBF and ASL CBF maps and coded as ROI (1), ROI (2) and ROI (3). All three ROI measurements were averaged among themselves and called ROI (mean). ROIs were placed in the same regions in each map, and another ROI measurement was made from the unaffected region in the opposite white matter of this area or from the opposite normal brain parenchyma. This ROI measurement was called ROI (r). The normalized value was obtained by proportioning ROI (mean) and ROI (r) (ROI (mean)/ROI (r)) and was used as a reference value for comparison purposes.

The suitability of these values to normal distribution was examined with histogram and Kolmogorov-Smirnov test. Spearman correlation test was performed for relationships between variables. Correlation coefficient 0.05–0.30 was accepted as low or insignificant correlation, 0.30–0.40 as low moderate correlation, 0.40–0.60 as moderate correlation, 0.60–0.70 as good correlation, 0.70–0.75 as very good correlation, and 0.75–1.00 as excellent correlation. Friedman’s two-way analysis of variance was applied to investigate whether there was a difference between the parameters in the three normalized values. Mann Whitney U test was used to see whether these normalized values differed between results.

The cases were divided into two groups: those with RRT and those with TRC, according to follow-up and clinical findings. The diagnostic decision-making features of the normalized values were examined by Receiver Operating Characteristics (ROC) curve analysis. In the presence of significant limit values, the sensitivity, specificity, positive predictive and negative predictive values of these limits were calculated. The cut off values are defined by the point closest to the top left corner. In the evaluation of the area under the curve (AUC), cases where the type 1 error level is below 5% are interpreted as the diagnostic value of the test being statistically significant. In the visual evaluation of the maps, the inter-reader agreement in distinguishing between the presence of TRC (0) and RRT (1) and the readers’ agreement with the results were evaluated with Kappa tests. Kappa score between 0 and 0.19 was defined as no agreement; between 0.20 and 0.39 as poor agreement; between 0.40 and 0.59 as moderate agreement; between 0.60 and 0.79 as good agreement; and between 0.80 and 1.00 as a perfect fit. For all statistics, a *p*-value of less than 0.05 was determined to be significant.

## Results

Of the 90 patients included in the study, 42 were women and 48 were men, and their average age was 53.4 years (20–81). Forty six patients had metastatic tumors, 43 patients had high-grade glial tumors, and one patient had lymphoma. According to the patients’ previous and follow-up MRIs and clinical findings, 47 patients had RRT and 43 patients had TRC.

In the quantitative evaluation, DSC nCBV was determined as 3.37 (min: 0.06-max: 43.14, 95% CI 1.81–4.73), DSC nCBF was 1.61 (min: 0.12-max: 9.21, 95% CI 1.27–1.95) and ASL nCBF was 2.02 (min: 0.13-max: 22.88, 95% CI 1.35–2.70) for all patients (Table 3).

**Table 3:** Values of TRC, RRT, DSC nCBV, DSC nCBF and ASL nCBF of the patients participating in the study according to tumor type

	Number n(%)	TRC n(%)	RRT n(%)	DSC nCBV	DSC nCBF	ASL nCBF
High-grade glial tumor	43 (47.8)	20 (22.2)	23(25.6)	3.86	1.95	2.69
Metastasis	46 (51.1)	22 (24.4)	24(26.7)	2.78	1.33	1.43
Lymphoma	1(1.1)	1 (1.1)	0 (0)	0.32	0.26	0.586
Total	90 (100)	43(47.8)	47 (52.2)	3.27	1.61	2.02

Uncorrected values of CBF and CBV were used in the study. Normalized values of all three parameters were used only in comparisons. In the nonparametric test performed to test the correlation of normalized values with each other, the DSC nCBV value was found to be highly correlated with the DSC nCBF value (r: 0.94, *p* < 0.001) and ASL nCBF (r: 0.75, *p* < 0.001). Similarly, ASL nCBF was highly correlated with DSC nCBF (r: 0.79 *p* < 0.01).

**Fig. 3:**
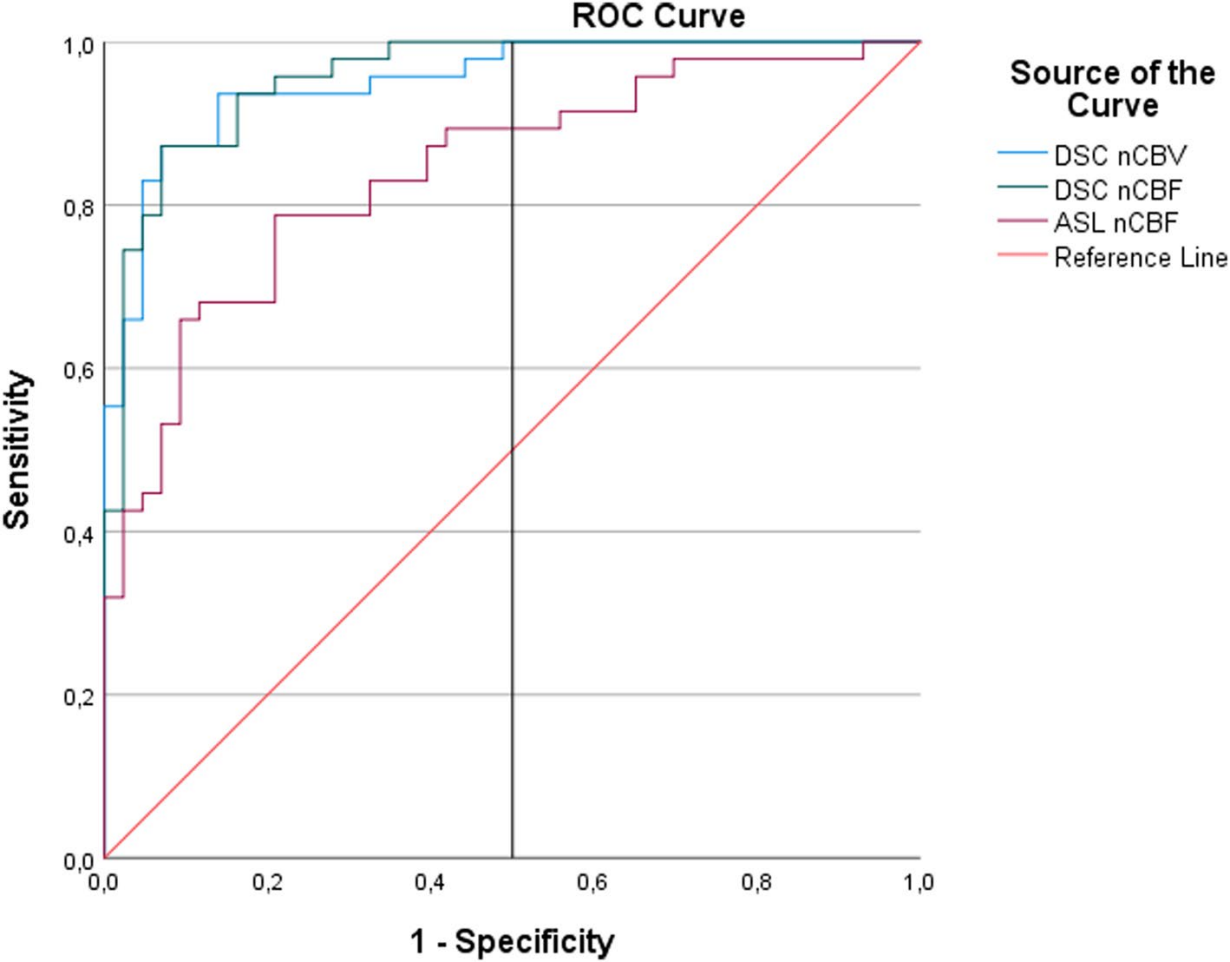
ROC Curve created with normalized values in cases with RRT

In the Friedman two-way analysis of variance conducted for the similarity of normalized numerical values, it was observed that the normalized numerical values were close for ASL nCBF and DSC nCBF (1.86 and 1.71, respectively), but DSC nCBV differed from both (2.43) (Table 4). ROC curve and AUC for normalized values were obtained in cases with RRT. In the ROC curve, DSC nCBV, nCBF and ASL nCBF values are above the reference line and can be used diagnostically (Fig. 3).

**Table 4:** Two-way comparison of the difference between normalized values

Example 1 - Example 2	Differences	Standard Error	P**
DSC nCBF-ASL nCBF	−0.14	0.14	0.99
DSC nCBF-DSC nCBV	0.72	0.14	< 0.001*
ASL nCBF-DSC nCBV	0.57	0.14	< 0.001*

*Significant difference between DSC nCBV and ASL nCBF and DSC nCBF

**p value corrected with Bonferroni

When ROC curve parameters were evaluated, cut-off values were determined as 1.211 for DSC nCBV (93% sensitivity, 82% specificity), 0.896 for DSC nCBF (93% sensitivity, 82% specificity), and 0.829 for ASL nCBF (78% sensitivity, 75% specificity). When the AUC values of DSC nCBV (AUC: 0.95, *p* < 0.001), DSC nCBF (AUC: 0.95, *p* < 0.001) and ASL nCBF (AUC: 0.84, *p* < 0.001) were compared, it was observed that DSC nCBV and DSC nCBF could make a very high rate of accurate diagnosis, and ASL nCBF could make a high rate of accurate diagnosis (Table 5).

**Table 5:** Results of receiver operating characteristics (ROC) curve analysis of DSC CBV, DSC CBF and ASL CBF values in the tumor periphery

Parameters	AUC	95% CI	Cutoff	Sensitivity	Specificity	*P* value
DSC r CBV	0.95	0.91–0.99	1.211	93.0	82.0	< 0.001
DSC r CBF	0.95	0.91–0.99	0.896	93.0	82.0	< 0.001
ASL r CBF	0.84	0.75–0.92	0.829	78.0	75.0	< 0.001

In TRC cases, the mean values for DSC nCBV, DSC nCBF and ASL nCBF were determined as 0.773 (min: 0.062-max: 2.554), 0.603 (min: 0.127-max: 2.143) and 0.709 (min: 0.134-max: 3.336), respectively. In RRT cases, the mean values for DSC nCBV, DSC nCBF and ASL nCBF were found to be 5.564 (min: 0.572-max: 43.145), 2.543 (min: 0.674-max: 9.212) and 3.238 (min: 0.178-max: 22.885), respectively. It was observed that all three normalized values showed a significant difference between the TRC and RRT groups (*p* < 0.001) (Table 6).

**Table 6:** Numerical data of all three parameters according to the result distribution and differences of normalized values ​​according to the results with Mann Whitney U test

		DSC nCBV	DSC nCBF	ASL nCBF
TCR	Mean	0.773 ± 0.58	0.603 ± 0.40	0.709 ± 0.61
	Median	0.561 (0.062–2.554)	0.492 (0.127–2.143)	0.534 (0.134–3.336)
RRT	Mean	5.564 ± 9.07	2.543 ± 1.77	3.238 ± 4.06
	Median	2.85 (0.572–43.145)	1.90 (0.674–9.212)	1.76 (0.178–22.885)
Mann Whitney	**Z:**	**−7.363**	**−7.452**	**−5.553**
U Test	**p:**	**< 0.001**	**< 0.001**	**< 0.001**

^**Z**^Mann Whitney U test

For qualitative evaluation (visual evaluation), Kappa agreement tests were performed between two separate readers who evaluated all three maps. Inter-observer agreement was found to be good for ASL CBF (0.714), good for DSC CBF (0.790), and excellent for DSC CBV (0.822) (Table 7). Observer agreement with the results was also evaluated. In the first observer, good agreement was found in ASL CBF (0.626, 70% sensitive, 93% specific), in DSC CBF (0.713, 76% sensitive, 95% specific), and in DSC CBV (0.755, 87% sensitive - 88% specific). In the second observer, moderate agreement was found in ASL CBF (0.584, 61% sensitive, 97% specific) and DSC CBF (0.649, 65% sensitive, 100% specific), and excellent agreement in DSC CBV (0.800, 89% sensitive, 90% specific) (Table 8).

**Table 7:** Observers’ compliance with the results

Maps/Results			Results	
			TRC	RRT
DSC CBV	TRC	Observer 1	38	6
		Observer 2	39	5
	RRT	Observer 1	5	41
		Observer 2 Interobserver reliability^a^	4 0.822	42
DSC CBF	TRC	Observer 1	41	11
		Observer 2	43	16
	RRT	Observer 1	2	36
		Observer 2	0	31
ASL CBF	TRC	Observer 1	40	14
		Observer 2 Interobserver reliability^a^	42 0.790	18
	RRT	Observer 1	3	33
		Observer 2 Interobserver reliability^a^	1 0.714	29

^a^Kappa Statistics

**Table 8:** Sensitivity/specificity ratios of observers according to results

Map/ sensitivity-specificity		Sensitivity (%)	Specificity (%)
DSC CBV	Observer 1	87	88
	Observer 2	89	90
DSC CBF	Observer 1	76	95
	Observer 2	65	100
ASL CBF	Observer 1	70	93
	Observer 2	61	97

## Discussion

The aim of this retrospective study was to qualitatively and quantitatively compare the perfusion values obtained from ASL with DSC MRI in patients treated for malignant brain tumor and to evaluate the response to treatment. It was observed that ASL nCBF values correlated very well with DSC nCBV and perfectly with DSC nCBF. It was determined that the ASL nCBF, DSC nCBV and DSC nCBF values of RRT were statistically and significantly higher than the ASL nCBF, DSC nCBV and DSC nCBF values of TRC.

Histopathological confirmation with biopsy is often required for definitive diagnosis of brain tumors. However, biopsy is invasive and challenging for some areas [21]. The treatment response criteria of the RANO study group guide the clinical examination and can help in the evaluation of perfusion maps [22]. Recently, with developing MRI techniques, new non-invasive imaging methods have been used to guide treatment plans by using physiological features of different tumors such as cellularity, oxygenation, vascularity and microstructure [23–25]. In DSC, which is based on T2-weighted imaging, a parallel imaging scan is required to cover the entire brain [26]. DSC is routinely used to differentiate low- and high-grade tumors and tumor recurrence and treatment-related changes. DSC provides perfusion parameters that correlate with histological structure such as rCBV and rCBF. However, its disadvantages include that it requires contrast injection and may cause a decrease in CBV values due to contrast material extravasation. ASL provides absolute CBF values by using an endogenous tracker [27, 28]. ASL perfusion is a method that is developing day by day and can obtain absolute CBF value noninvasively and without the use of contrast [24].

In a study conducted on 30 patients with a history of treatment for GBM (1,5 T, pASL), ASL nCBF values were determined to be higher than DSC nCBV values. No difference was observed between patients with and without tumor recurrence. Researchers have suggested that since the single-PLD ASL method was used, it may cause bias in the evaluation [16]. Ye et al. [29] documented that in patients treated for glioma, ASL nCBF values were higher than DSC nCBV and that there was a significant difference in both parameters between the recurrent gliomas and radiation necrosis groups. On the contrary, in the study conducted by Manning et al. [30], it was found that ASL nCBF values were smaller than DSC nCBV, but similar to DSC nCBF, and the values in the progression group were statistically significantly higher for all three parameters. In ASL examination of 26 patients with a history of treatment for GBM (1,5 T, pASL), Seeger et al. [31] did not observe any difference between the group with tumor recurrence and stable disease (2.41 ± 1.3 vs 1.66 ± 0.5, respectively). In the same study, it was reported that DSC nCBF and DSC nCBV values were higher than ASL nCBF, and that there was a significant difference in DSC-related parameters between tumor recurrence and radiation necrosis groups. Jovanovic et al. [32] reported that DSC nCBV values were similar to other studies and ASL nCBF values were lower compared to other studies.

In our study, uncorrected DSC nCBV values were found to be higher than both ASL nCBF and uncorrected DSC nCBF and previous study results. In the study conducted by Arisawa et al. [33] using histogram analysis in benign and malignant glial tumors, it was stated that the maximum values were found to be higher in DSC nCBV, and they suggested that this may be due to the inability to completely remove pixels belonging to vascular structures in the histogram analysis. Moreover, in a study conducted by Hashido et al. [28], using radiomics for comparison, similar results were obtained. Therefore, it was thought that the use of manual measurement in our study did not affect the results. Previous studies have suggested that taking the reference value from the white matter and low spatial resolution may cause the ASL nCBF measurement to be smaller than normal [34, 35]. In our study, manual measurements, not using corrected values, reference values being taken from white matter, and heterogeneous malignant tumor histologies may be responsible for these results. Also, in our study, it was observed that the DSC nCBV, DSC nCBF and ASL nCBF values of RRT patients were significantly higher than those of TRC patients. These results of our study confirm the results of previous studies.

In previous studies evaluating brain tumor perfusion, the correlation of diagnostic comparison parameters was also evaluated. Ata et al. (1,5 T) reported an excellent correlation (*r* = 0.91, *P* < 0.001) between DSC nCBF and nCBV values [36]. In the linear correlation analysis of Jovanovic et al., a very good correlation was found between ASL nCBF and DSC nCBV (r: 0.733) [26]. Lavrova et al. [37] determined that there was a moderate correlation (r: 0.60–0.67) between ASL nCBF and uncorrected DSC nCBV, and a good correlation (r: 0.72–0.78) between ASL CBF and corrected nCBV. Researchers also observed a strong correlation in contrasted gliomas (r: 0.65–0.80) and low correlation in non-contrasted gliomas (r: 0.58–0.73) and brain metastases (r: 0.14–0.40) when they looked at disease specificities [37]. Xu et al. [38] found an excellent correlation between DSC nCBF-nCBV, ASL nCBF-DSC nCBF and ASL nCBF-DSC nCBV in the quantitative evaluation of patients receiving treatment for glial tumors. Rau et al. [9] determined a moderate correlation between DSC nCBF and nCBV in high-grade gliomas. However, they did not observe a statistically significant correlation between ASL nCBF and DSC parameters. In another study, it was determined that there was a close linear correlation between normalized perfusion values obtained from ASL and DSC [39]**.** In our study, it was observed that uncorrected DSC nCBF showed excellent correlation with both uncorrected DSC nCBV and ASL nCBF. Additionally, uncorrected DSC nCBV showed a very good correlation with ASL nCBF. The different correlation rates determined in the studies may be related to the method used, the number of patients and the histological structure of the tumor.

ROC curve analysis was performed to evaluate the diagnostic performance of the parameters in the applied methods. Jovanovic et al. [32] determined that when the ROC curve was used, DSC nCBV had 100% sensitivity and specificity (AUC = 1.000; *p* < 0.001) when the cut-off value was 2.89. They also observed that ASL had 100% sensitivity and 73.7% specificity when the cut-off value was 0.995, and 92.3% sensitivity and 92.9% specificity when the cut-off value was 1.02 (AUC = 0.967; *p* < 0.001). In another study, the cut-off value in ROC analysis was determined as 2.18 for ASL nCBF (84.6% specificity, 53.9% sensitivity, AUC: 69%), and the cut-off value for DSC nCBV was determined as 2.24 (84.6% specificity, 77.3% sensitivity, AUC: 80%) [31]. In their ROC curve analysis, Xu et al. [38] found the cut-off values to be 1.11 (AUC 0.88, sensitivity 100%, specificity 75%) for ASL nCBF, 2.36 (AUC 0.86, sensitivity 70%, specificity 91%) for DSC nCBF and 3.64 (AUC 0.82, sensitivity 58%, specificity 100%) for DSC nCBV. Lavrova et al. [37] measured AUC values of 0.73–0.80 for ASL nCBF and DSC nCBV. While researchers determined AUC values of 0.78 and 0.77–0.80 for ASL nCBF and DSC nCBV in enhancing gliomas, they found them to be 0.72 for ASL nCBF and 0.87–0.93 for DSC nCBV in brain metastases. Özsunar et al. [16] determined that the ASL technique exhibited higher sensitivity and specificity compared to DSC in detecting recurrent tumors (88% sensitivity-89% specificity vs. 86% sensitivity-70% specificity, respectively). Rau et al. [9] found DSC nCBV to be superior to DSC nCBF and ASL nCBF in predicting recurrence (AUC: 0.71, AUC: 0.59 and AUC: 0.58, respectively). In another study, AUC values for ASL nCBF, DSC nCBF and DSC nCBV were determined as 0.95, 0.86 and 0.89, respectively [30]. In the evaluation of patients receiving treatment for GBM, Choi et al. [40] determined that the diagnostic accuracy of DSC when used alone was 75.8%, and with the addition of ASL perfusion, the accuracy rate increased to 88.7%.

In our study, according to the ROC curve analysis results, the cut-off values were determined as 1.211 (93% sensitivity, 82% specificity) for uncorrected DSC nCBV, 0.896 for (93% sensitivity, 82% specificity) uncorrected DSC nCBF and 0.829 (78% sensitivity, 75% specificity) for ASL nCBF. ASL nCBF, DSC nCBF and DSC nCBV cut-off values in our study were observed to be lower than previous studies [28, 30–38]. Additionally, the detection of lower cut-off values in all parameters (ASL nCBF, DSC nCBV, DSC nCBF) compared to the literature has been attributed to differences in the number of patients, the larger size of the group with TBD, the lower minimum values compared to the literature, the lower measurements taken from the operation cavity without recurrence, or the use of low-area ROI in these regions, resulting in lower minimum values compared to the literature.

In our study, the sensitivity and specificity values for uncorrected DSC nCBV were generally found to be higher when compared to the corrected DSC nCBV values reported in the literature [28, 30–38]. As for uncorrected DSC nCBF, the sensitivity in our study was higher compared to the literature, while the specificity was similar or lower.and corrected DSC nCBF [30, 38]. It has been considered that the higher sensitivity detected in DSC nCBV and DSC nCBF compared to the literature may also be attributed to the use of uncorrected values. In comparison to the literature, the sensitivity of ASL nCBF is lower, but its specificity is higher than some studies [16, 31, 32, 38]. Our ASL imaging techniques are similar to only one study among these [38]. In a study comparing perfusions in primary and secondary brain tumors [37], no significant correlation was found between ASL and DSC perfusions in metastases, and a decrease in ASL nCBF AUC values compared to corrected DSC nCBV was observed in cases with brain metastases. The discrepancy in sensitivity and specificity between our study and Xu et al. [38] was thought to be possibly due to the high number of metastases in our study.

In our study, AUC values for ASL nCBF, uncorrected DSC nCBF and uncorrected DSC nCBV were determined as 0.84, 0.95 and 0.95, respectively. Our study found that diagnostically, all three parameters can be used in the differentiation between TBD (treatment-related changes) and NRT (non-residual tumor), but uncorrected DSC nCBV and DSC nCBF are superior. Similarly to literature findings [9, 32, 37], studies have correlated with our results, showing higher AUC values for DSC nCBV compared to ASL nCBF. Some studies have reported higher AUC values for ASL nCBF [30, 38]. However, Xu et al. did not find the difference in AUC values to be statistically significant [38]. Manning et al. conducted a study similar to ours (3 T, pCASL), with higher AUC values for ASL nCBF [30]. The differences could be attributed to Manning et al. having only GBM operative cases, a lower number of pseudoprogression cases (n:7), and a smaller overall patient population (n:32), as well as the use of different ASL reading techniques [30].

In our study, in qualitative assessment, it was observed that two readers could evaluate DSC CBV more consistently with each other and with the results compared to the other two parameters. The sensitivity was found to be higher compared to other techniques. In ASL CBF and DSC CBF maps, higher specificity was observed. In contrast to previous studies in the literature [16, 36], our study found that the specificity of ASL CBF may be higher than its sensitivity. Jarnum et al., compared the 3D pCASL method with the DSC method in terms of susceptibility artifacts and noted that the artifacts were statistically significantly less in ASL. In this case, they suggested that using FSE instead of GRE EPI could be effective [41]. In the study conducted by Manning et al., the 3D pCASL technique was employed, and signal acquisition was performed using the spiral Fast Spin Echo (FSE) method. This study also indicates that sensitivity artifacts were less in ASL compared to DSC [30]. The difference in sensitivity in our qualitative assessment compared to the literature may be attributed to the unclear efficacy of ASL in metastatic lesions, the continued inadequacy of SNR despite the preference for patients with tumor could be evaluated GRASE pCASL, and the potential errors in labeling due to susceptibility artifacts in treated cases. The higher specificity compared to sensitivity, contrary to studies in the literature, may be linked to the sensitivity to artifacts in the GRASE reading technique, resulting in the elimination of false positives, as suggested by Maral et al. [42]. Similarly, the more pronounced susceptibility artifacts in the GRASE method compared to FSE may explain the differences between our study and the quantitative evaluations and specificity-sensitivity levels in the literature [30]. DSC CBF also showed similar sensitivity and specificity in visual assessment compared to ASL CBF.

ROI measurements, an operator-dependent method, are used in DSC-MRI data analysis [36]. Tumoral CBV measurement remains operator dependent and highly subjective [43]. In a multicenter study, reproducibility and repeatability were reported to be less than 10 and 50%, respectively [44]. A good review process can help evaluate perfusion measurements more objectively. For this reason, it has also been recommended that DSC-MRI data be evaluated by two experienced observers [45]. In our study, DSC and ASL results were evaluated separately by two observers. Interobserver agreement was good for ASL CBF (0.714), good for DSC CBF (0.790), and excellent for DSC CBV (0.822). Additionally, intra-observer agreement was generally good.

The strengths of our study were that the patient group is larger compared to previous studies, there were different groups with numerically similar distribution in the lesion groups, patients with tumor could be evaluated with 3D GRASE pCASL, and there was no bias in favor of ASL in visual evaluation due to sufficient time between readings. There are some limitations in our study. First, corrected values were not used in DSC measurements and patient results were obtained through follow-up and control evaluations rather than histopathologically. Second, the treatment-related changes group was not divided into radiation necrosis/pseudoprogression and stable disease during follow-up. Third, the values taken from the operation cavity were found to be low in cases that underwent surgery and did not have significant radiation necrosis. Lastly, the limitation is that the ROI sizes used are not standard due to different lesion sizes and the study was conducted retrospectively.

In conclusion, we observed that the DSC nCBF value was perfectly correlated with DSC nCBV and ASL nCBF, and DSC nCBV was very well correlated with ASL nCBF. DSC nCBV, DSC nCBF and ASL nCBF values of RRT were significantly higher than those of TRC. It was determined that all three parameters DSC nCBV, DSC nCBF and ASL nCBF were usable from a diagnostic perspective, but the highest rate belonged to DSC nCBV. It was also observed that DSC CBV had higher sensitivity and ASL CBF had higher specificity. In visual evaluation, DSC CBV is the test that can be evaluated best among all three parameters. In the qualitative evaluation, ASL CBF was found to have low sensitivity and high specificity. Prospective studies with larger patient participation will make important contributions.

## Data Availability

The datasets used and/or analyzed during the current study are available from the corresponding author on reasonable request.

## Abbreviations

CNS: Central nervous system
RT: Radiotherapy
MRI: Magnetic resonance imaging
PWI: Perfusion-weighted imaging
CBF: Cerebral blood flow
CBV: Cerebral blood volume
nCBV: Normalized CBV
nCBF: Normalized CBF
MTT: Mean transit time
DCE: Dynamic contrast-enhanced
DSC: Dynamic susceptibility contrast-enhanced
ASL: Arterial spin labeling
TGSE: Turbo gradient spin echo
ROI: Region of interest
TRC: Treatment-related changes
RRT: Residual or recurrent tumor
ROC: Receiver operating characteristics
AUC: Area under the curve
GBM: Glioblastoma multiforme
GRASE: Gradient and spin echo
FSE: Fast spin echo
CI: Confidence interval
